# Relating GPCRs pharmacological space based on ligands chemical similarities

**DOI:** 10.1186/1758-2946-5-S1-P26

**Published:** 2013-03-22

**Authors:** Alexios Koutsoukas, Rubben Torella, George Drakakis, Andreas Bender, Robert C Glen

**Affiliations:** 1 Unilever Centre for Molecular Informatics, Department of Chemistry, Lensfield Road, University of Cambridge, Cambridge, CB2 1EW, UK

## 

G protein-coupled receptors (GPCRs) are a major family of membrane receptors in eukaryotic cells and play a crucial role in various biological processes. They represent a family of protein targets with significant therapeutic value, and accordingly more than 30% of prescription drugs are GPCR ligands [1].

Extending previous attempts to map the pharmacological space solely based on ligand chemical similarity,[2,3] we in this work relate GPCRs pharmacological space by combining structure-activity data from ChEMBL and WOMBAT that covers 167 human GPCRs and 67k ligands. By including more information from the ligand side in our analysis than previous studies, we hence attempted to construct a more detailed map of the pharmacological space. A statistical approach similar to the "Similarity Ensemble Approach" (SEA)[2] was implemented to relate proteins based on the chemical similarity of their ligands, and to rank the significance of the resulting similarity scores. A prospective external validation dataset was then employed to confirm new relationship between ligands and different GPCRs, providing mechanistic evidence for observed side effects of drugs in the dataset.

The results of the study aim to contribute to a better understanding of the overlap of GPCRs in chemical space, and to the cross-reactivity observed even among distant biological targets, as defined by their sequence similarities[4] . Relevant applications range from understanding drug side effects to the design of drugs with a desired polypharmacological profile.
